# The Relationship Between Access to Natural Environmental Amenities and Obesity

**DOI:** 10.7759/cureus.377

**Published:** 2015-11-11

**Authors:** Benjamin Littenberg, Levi N Bonnell, Ayodelle S LeBruin, Derek A Lubetkin, Austin R Troy, Asim Zia

**Affiliations:** 1 General Internal Medicine Research, University of Vermont; 2 Department of Ophthalmology, University of Colorado School of Medicine; 3 Department of Planning and Design, University of Colorado Denver; 4 Community Development and Applied Economics, University of Vermont

**Keywords:** obesity, environment and public health, environmental epidemiology, data

## Abstract

Background

Various aspects of the environment are correlated with obesity. Most of the previous work in this area centers on the built environment. We sought to better understand the association of the natural environment with obesity.

Methods

We used the Natural Amenities Scale to characterize the attractiveness of 2,545 US counties based on access to open water, varied topography, and mild climate. We obtained the height, weight, age, sex, and address of adults from three different sources. The Departments of Motor Vehicles from seven US states provided over 38 million records. A web survey contributed 3,012 from 48 states and the District of Columbia. A clinical study of adults with diabetes from four states provided 974 more for a total of 38,159,046 analyzable records. We used logistic regression to model the association of obesity with natural amenities while controlling for age, sex, year of data collection, and various socioeconomic characteristics of the county.

Results

Natural amenities were inversely associated with obesity in all three populations. Over 20% of residents of low amenity areas were obese, but less than 10% of those living with the best natural amenities were obese.

Conclusions

The natural environment may affect health. Residing in areas with access to open water and a variety of topographic features as well as cool, dry summers and warm, sunny winters is associated with lower rates of obesity.

## Introduction

The environment is associated with health issues. For instance, obesity tends to be more common in the southern United States than New England or the Pacific Northwest [1]. Various aspects of the built environment, including the degree of development, the transportation network, and access to food distribution points, are associated with the prevalence of obesity [2]. The natural environment may also be a contributor to energy balance and obesity [3-4].

Lin, et al. used weather station records to estimate climate amenable for physical activity at the county level and linked them to telephone survey data from 2002. They reported lower body mass indices (BMI) among those counties with the most amenable climate after controlling for individual risk factors, road density, household income, and unemployment [4]. McGinn, et al. showed an association between physical activity and perceived measures of the natural environment, but not to objective measures [3].

Like the built environment [5], the natural environment is a multi-dimensional construct. Depending on context, it is characterized by factors such as topography, soils, hydrology, climate, vegetation, and wildlife. A subset of these features were combined to create the Natural Amenities Scale “based on the premise that people are drawn to areas with varied topography; lakes, ponds, or oceanfront; warm, sunny winters; and temperate, low-humidity summers” [6]. It was developed primarily to study rural migration patterns [7]. However, Jilcott, et al. used the Natural Amenities Scale to study the relationship between the environment and obesity in North Carolina [8]. Obesity was measured by the Behavioral Risk Factor Surveillance Survey conducted by random-digit telephone calls. They reported a negative correlation between amenities and obesity at the county level and presented some data showing that the effect may be mediated by physical activity. More recently, this group extended the work to include 3,106 counties in 48 states and again showed an inverse association between natural amenities and obesity [9].

We sought to expand on these analyses by examining the relationship between natural amenities and body mass measured at the individual level using data from three additional data sets.

## Materials and methods

We used data from over 38 million individuals to build regression models of the relationship between natural amenities and obesity while controlling for possible confounders. We anticipated that obesity would be less common in areas with the most attractive amenities. The null hypothesis was that obesity is not associated with natural amenities. We then re-examined the robustness of the model in two independent data sets that offer complementary strengths and weaknesses. 

### Data

Amenities and Other County-Level Descriptors

We used descriptors of counties from the Inter-university Consortium for Political and Social Research [10]. The data included the level of natural amenities for nearly all the counties in the United States from the ERS Natural Amenity Scale [6]. Developed by the Economic Research Service of the Department of Agriculture, this scale reflects measures of climate and topography that most people prefer: warm sunny winters, temperate dry summers, variation in topography, and access to rivers, streams, lakes, and oceans [7]. The data source does not include Alaska, Hawaii, Puerto Rico, or the Virgin Islands. Thirty-eight counties had values estimated from adjacent counties. The amenity scale is centered near zero (mean of 3,111 counties = 0.06, median = -0.13) and runs from -6.4 (Red Lake, MN) to +11.17 (Ventura, CA) with higher values representing more attractive amenities. The interquartile range runs from -1.42 to +1.10. The top ten counties are all in California. The ten lowest scoring counties include one from Indiana, three from North Dakota, and six from Minnesota.

The Inter-university Consortium for Political and Social Research data set also contains county-level estimates of the mean per capita personal income in 2005 from the US Department of Commerce (Bureau of Economic Analysis, Regional Economic Information System 1969-2005), the 2005 unemployment rate from the US Department of Labor (Bureau of Labor Statistics, Local Area Unemployment Statistics Program), the 2004 crime rate from data compiled by the Uniform Crime Reporting Program at the Federal Bureau of Investigation, and the land area, longitude, latitude, median age, percent of residents in various racial and ethnic groups, population, and number of housing units in 2005 from the US Census. Income was expressed in thousands of dollars per year.

Departments of Motor Vehicles (DMV) Data

We obtained the records of drivers’ licenses and non-driver identification cards from the states of Illinois, Maine, Michigan, Oregon, Texas, Vermont, and Washington, including date of issue, height, weight, age at the time of issuance, gender, and home address. The zip code of the home address was coded to a specific US county. If a zip code is associated with more than one county, it was assigned to the county with the highest proportion of residential addresses from that zip code [11]. We received 53,794,943 records from the Departments of Motor Vehicles of seven states and omitted 1,483,013 from before 1966, 13,232,186 because they applied to subjects under the age of 18, and 924,684 because they were missing one or more key variables, leaving 38,155,060 records from 2,524 counties in 48 states and the District of Columbia available for analysis (0.14% of DMV records contained a home address out of the state of issue).

The University of Vermont Committee on Human Subjects and the Colorado Multiple Institutional Review Board considered the data exempt from institutional review.

GeoMed

GeoMed is a web-based survey that recruited adults via social media and e-mail in 2014 and 2015. Respondents provided their height, weight, age, gender, race, education, physical activity level, general health, and home address. We used the same method of determining the county of each address as described for the DMV data above. Of 3,191 US residents who completed the survey at the time of analysis (data collection is ongoing at https://redcap.uvm.edu/redcap/surveys/index.php?s=AgCWxtoyMX), 42 were under age 18, and 137 had incomplete data, leaving 3,012 subjects representing 587 counties from 48 states and the District of Columbia for analysis. 

The survey was approved by the University of Vermont Committee on Human Subjects.

Vermont Diabetes Information System (VDIS)

The Vermont Diabetes Information System (VDIS) was a cluster-randomized trial of a decision support system in community primary care practices [12]. Study participants were patients receiving care for diabetes from 64 primary care practices in Vermont and adjacent Northern New York. Patients under 18 years, receiving their diabetes care from specialists, or with significant cognitive impairment per the judgment of the primary care provider, were excluded. The 7,412 VDIS subjects were contacted by telephone in random order until a sample of approximately 15% of the subjects from each practice agreed to participate in an in-person interview, including measurement of height using a portable stadiometer (SECA GmbH, Hamburg, Germany) and weight using a portable scale (Health O Meter LB Dial Scale HAP200KD-41, SunBeam, Inc., Purvis, MS). One thousand and two interviews took place between July 2003 and March 2005 [13]. Twenty-eight were missing data elements, leaving 974 analyzable subjects from 19 counties in Vermont, New York, New Hampshire, and Massachusetts.

The study was approved by the University of Vermont Committee on Human Subjects #14-207. 

### Analytic approach

We calculated the body mass index (BMI) for each subject as their weight in kilograms divided by their height in meters squared. Values of 30 kg/m^2^ or higher were classified as obese. We explored the relationship between the ERS Natural Amenity Scale and obesity graphically by constructing a non-parametric locally-weighted smoothing scatterplot (LOWESS) curve [14]. LOWESS curves do not require the *a priori *specification of a functional form, allowing them to serve as graphical descriptors of two-dimensional relationships. Because LOWESS is computationally intense, we applied it to a subset of the data consisting of 100,000 randomly selected DMV records. All other analyses used all available records.

We used logistic regression to assess the relationship between the amenity scale and obesity (coded as 1 for obese subjects and 0 for non-obese subjects) while controlling for potentially confounding covariates. Individual-level covariates included age, gender, and year of data collection (to control for secular trends in BMI). County-level covariates included latitude and longitude (to control for regional variations in obesity), various social and economic characteristics of the community (median age, percent of residents in each of seven categories of race and ethnicity, unemployment rate, per capita income), and markers of development (housing density and population density).

We created a logistic model, including all the potential confounders using the DMV data. We eliminated potential confounders in a backward stepwise fashion starting with the highest P-value until all remaining predictors were associated with obesity with P<0.05. We then built three separate logistic models for each of the three data sources using the variables that were retained in the reduced model. In this way, all three models were comparable in terms of covariates. We adjusted all regressions for clustering of individual subjects within counties using the robust “sandwich estimator” method [15]. We calculated 95% confidence intervals (CI) for each parameter and considered a two-tailed P<0.05 as evidence of statistical significance.

## Results

The prevalence of obesity was 22% among the 38,155,060 individuals in the DMV data, 21% in the GeoMed online survey, and 67% among the diabetic patients from the VDIS (Table 1). The VDIS population was also older and came from counties that had less racial diversity, lower personal incomes, and much lower population and housing densities than the other sources. The GeoMed respondents were younger, but otherwise generally similar to the DMV data. The VDIS data also had lower Natural Amenity Scores. In fact, none of the VDIS participants lived in counties with scores above the mean of either of the other two groups. The distribution of land types is quite different across the three sources with most of the DMV and GeoMed data coming from the plains with the majority of VDIS data coming from highland regions.

**Table 1:** Characteristics of the three populations DMV = Department of Motor Vehicles data; GeoMed = GeoMed on-line survey data; VDIS = Vermont Diabetes Information System data; SD = standard deviation; D.C. = District of Columbia

	DMV		GeoMed		VDIS	
	Mean	SD	Mean	SD	Mean	SD
Individuals (n)	38,155,060		3,012		974	
Obese	22.1%		21.1%		67.4%	
Sex (male)	49.8%		45.9%		45.6%	
Age (y)	40.2	18.2	34.4	13.5	64.8	12.0
Year of data collection	2004	12	2014	0	2004	0
Height (m)	1.7	0.1	1.7	0.1	1.7	0.1
Weight (kg)	77.5	18.8	78.7	22.5	92.4	21.6
Counties (n)	2,524		587		19	
ERS Natural Amenity Scale	0.6	2.4	0.8	2.8	-0.6	0.7
Longitude (degrees)	-97.7	13.7	-88.2	16.0	-73.4	0.9
Latitude (degrees)	38.6	6.7	39.7	4.7	44.1	0.7
Median age of total resident population (y)	35.1	3.4	36.1	3.2	38.9	2.6
Percent of resident population:						
White	83.0	10.8	80.3	15.4	96.1	2.6
Black	10.7	9.8	12.9	14.7	1.3	1.6
American Indian or Alaska native	0.8	0.9	0.8	1.8	0.8	1.2
Asian	3.9	3.4	4.3	4.6	0.8	0.6
Native Hawaiian or Pacific islander	0.2	0.2	0.1	0.2	0.0	0.0
Two or more races	1.5	0.9	1.6	0.7	0.9	0.4
Hispanic	20.2	20.5	10.5	12.1	1.4	0.9
Unemployment rate	5.7	1.2	4.7	1.3	4.1	1.1
Per capita personal income ('000 $)	34.2	7.9	37.9	10.1	30.1	4.7
Housing units per hectare	1.9	2.5	4.7	15.9	0.1	0.1
Population density per hectare	4.9	6.4	10.6	32.7	0.3	0.3
Mean temperature in January	36.4	11.9	31.8	12.3	18.1	1.9
Mean hours of sunlight in January	127.5	42.7	146.6	37.2	121.5	11.8
Mean temperature in July	76.0	7.4	74.3	5.2	69.5	1.2
Mean relative humidity in July	51.3	10.5	58.9	13.0	63.9	1.0
Topography:						
Plains	61.1%		40.9%		0%	
Tablelands	6.6%		9.4%		0%	
High Plains	5.1%		6.9%		16.7%	
Open Highlands	10.2%		26.9%		73.0%	
Hills and Mountains	17.0%		16.0%		10.3%	
Percent of area covered by water	10.3	16.2	9.6	13.3	4.9	5.8
States (n)	48 plus D.C.		48 plus D.C.		4	

**Figure 1:**
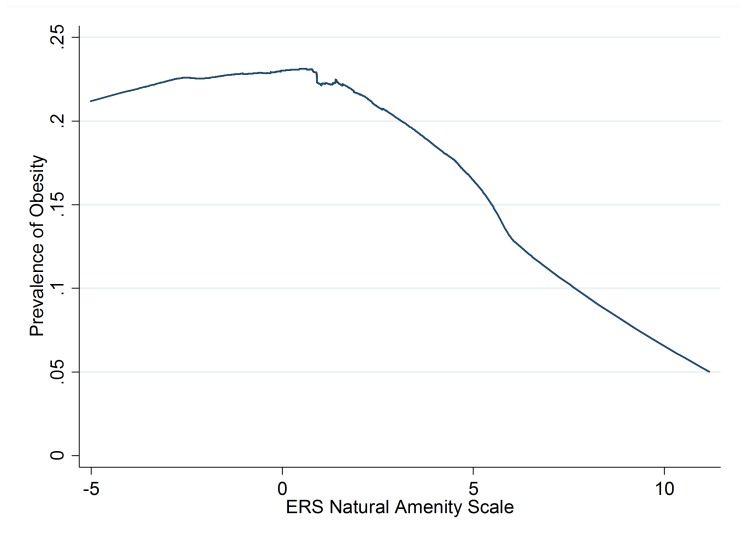
Relationship of Obesity to Natural Amenities Locally-weighted smoothing scatterplot (LOWESS) curve based on a randomly selected subset of 100,000 DMV records.

Obesity in the DMV data was associated with amenities in the LOWESS analysis. The prevalence of obesity was above 20% among subjects living in counties with ERS Natural Amenities Scale scores below 3 and below 10% where scores were above 7 (Figure 1).

In multivariate logistic regression with the full set of confounders, the ERS Natural Amenities Scale was associated with obesity with an odds ratio (OR) of 0.96 per point (95% CI = 0.95, 0.98; P < 0.001) (Table 2). After stepwise reduction, the reduced model included subject age and year of measurement as well as county-level latitude, median age, unemployment rate, and median income as covariates. Obesity remained significantly associated with the ERS Natural Amenity Scale (OR = 0.97; 95% CI = 0.96, 0.98; P < 0.001). Given a baseline prevalence of obesity of 22%, a one point difference in the Natural Amenity Scale is associated with a 0.6% change in the prevalence of obesity (Consider that Champaign in Central Illinois scores -4.5, Odessa, TX scores +2.5, Seattle, WA +4.5, and Clallam County, Washington on the Olympic Peninsula scores +6.5). Older subjects, residents of more southern counties and residents of counties with older median ages and higher unemployment had higher rates of obesity. Residents of counties with higher income had lower rates of obesity.  

**Table 2:** Logistic regression of Natural Amenity Scale on obesity in the DMV data OR = odds ratio; CI = confidence interval

	Full Model				Reduced Model			
	OR	t	*P*	95% CI	OR	t	*P*	95% CI
ERS Natural Amenity Scale	0.964	-4.45	<0.001	0.948, 0.980	0.968	-6.69	<0.001	0.959, 0.977
Individual covariates								
Age (y)	1.007	12.18	<0.001	1.006, 1.008	1.007	11.99	<0.001	1.006, 1.008
Sex (male)	1.061	1.89	0.059	0.998, 1.128				
Year of data collection	0.987	-21.17	<0.001	0.986, 0.988	0.986	-20.71	<0.001	0.985, 0.987
County-level covariates								
Longitude (degrees)	1.001	0.86	0.388	0.998, 1.004				
Latitude (degrees)	0.982	-6.74	<0.001	0.977, 0.987	0.979	-9.90	<0.001	0.975, 0.983
Median age of total resident population (y)	1.013	3.32	0.001	1.005, 1.021	1.010	3.87	<0.001	1.005, 1.016
Percent of resident population:								
White	1.750	0.40	0.689	0.113, 27.17				
Black	1.757	0.40	0.687	0.113, 27.27				
American Indian or Alaska native	1.732	0.39	0.694	0.112, 26.87				
Asian	1.689	0.37	0.708	0.109, 26.25				
Native Hawaiian or Pacific islander	1.813	0.43	0.670	0.118, 27.90				
Two or more races	1.893	0.46	0.649	0.121, 29.51				
Hispanic	1.003	3.21	0.001	1.001, 1.005				
Unemployment rate (%)	1.057	5.10	<0.001	1.035, 1.080	1.067	6.32	<0.001	1.046, 1.089
Per capita personal income ('000 $)	0.992	-3.22	0.001	0.987, 0.997	0.982	-9.13	<0.001	0.979, 0.986
Housing units per hectare	1.094	1.88	0.061	0.996, 1.201				
Population density per hectare	0.959	-2.13	0.034	0.923, 0.997				
Number of subjects	38,155,060				38,155,060			
Number of counties	2,524				2,524			
R^2^	0.02				0.02			
*P*	<0.001				<0.001			

To assess the possibility of long-term secular changes not captured by including the year of measurement in the model, we divided the data into five subsets based on the decade of measurement (1966-1975, 1976-1985, etc.*) *and applied the same reduced model to each subset. There was very little change in the adjusted OR on Natural Amenity Scale with values ranging from 0.96 to 0.98.

In the separate analyses, the subjects from the DMV data and the GeoMed survey had very similar results while the model based on the VDIS was somewhat different. Each of the ORs in the GeoMed data were similar to those in the DMV model in direction and magnitude, except that year of data collection county-level per capita personal income were no longer significantly associated with obesity. The VDIS model had notably different odds ratios on each of the predictors, including a change in direction for the effect of subject age, year of data collection, latitude, unemployment rate, and personal income (Tables 3-4).

**Table 3:** Logistic regression of Natural Amenity Scale on obesity in the GeoMed data OR = odds ratio; CI = confidence interval

	OR	t	*P*	95% CI
ERS Natural Amenity Scale	0.951	-2.54	0.011	0.914, 0.988
Age (y)	1.016	4.40	<0.001	1.009, 1.024
Year of data collection	1.000	0.01	0.999	1.000, 1.000
Latitude (degrees)	0.942	-3.88	<0.001	0.914, 0.971
Median age of total resident population (y)	1.067	3.70	<0.001	1.031, 1.105
Unemployment rate	1.033	0.53	0.596	0.916, 1.166
Per capita personal income ('000 $)	0.979	-3.95	<0.001	0.969, 0.989
Number of subjects	3,012			
R^2^	0.03			
*P*	<0.001			

**Table 4:** Logistic regression of Natural Amenity Scale on obesity in the VDIS data OR = odds ratio; CI = confidence interval

	OR	t	*P*	95% CI
ERS Natural Amenity Scale	0.655	-2.24	0.025	0.452, 0.948
Age (y)	0.952	-7.59	<0.001	0.940, 0.964
Year of data collection	1.094	0.85	0.394	0.889, 1.347
Latitude (degrees)	1.996	5.03	<0.001	1.525, 2.613
Median age of total resident population (y)	1.170	3.86	<0.001	1.080, 1.266
Unemployment rate	0.968	-0.23	0.820	0.732, 1.281
Per capita personal income ('000 $)	1.033	1.76	0.079	0.996, 1.072
Number of subjects	974			
R^2^	0.06			
*P*	<0.001			

## Discussion

### Major findings

The natural environment, including climate and topography, is associated with obesity across a broad range of populations and landscapes in America. The relationships seen here among licensed drivers, holders of non-driving identity cards, respondents to a web-based survey, and patients with diabetes are similar to those shown using the data collected by random-digit telephone surveys [8-9, 16].

The multivariate analysis controlled for age, sex, temporal trends, county geographic position, demographics, economics, and degree of development, indicating that these factors are not confounding the relationship between natural amenities and obesity. Although the three data sources include quite different groups of subjects, they all show the same basic relationship of a lower prevalence of obesity at higher levels of natural amenities.

The differences among the three models warrant some discussion. The GeoMed survey and VDIS each had limited ranges of survey years, explaining why the year of data collection is not significant in those two models. Otherwise, the model based on the GeoMed survey is quite similar to that derived from the DMV data, except for differences that may be explained by the much smaller sample size. The VDIS survey, on the other hand, shows a much stronger association with the Natural Amenity Scale and different directions for most of the covariates. We attribute the differences in the models to the very different nature of the population. They were all chronically ill with a disease that is often caused by obesity and that may limit physical activity if the patient develops complications, such as painful neuropathy of the feet or heart disease. The VDIS was conducted in a relatively restricted geographic region of just 19 contiguous counties, nearly all of which are extremely rural. In spite of these factors, which might tend to limit the effect of the environment on caloric expenditure, natural amenities appear protective in this population as well.

### Implications

Although these data cannot conclusively demonstrate causality, one possible mechanism for the role of the natural environment on obesity includes promotion of a healthier lifestyle through outdoor recreation. Other aspects of lifestyle, such as access to commercially-prepared calorie-dense foods and engagement in more physically demanding occupations, are also possible.

Analyses of individual correlates of health status can help to inform prescription for personal health. In other words, some people may choose to move to locations with amenities in the hope that it will have health benefits. Likewise, analyses of the built environment can guide public policy about urban form, building codes, and development patterns. However, the environment characteristic included in the Natural Amenities Scale generally cannot be modified to suit human needs. Nonetheless, the insights from analyses of natural correlates to health can be useful. First, they lead us to explore the particular aspects of the environment that are likely to be causative and help explain the mechanisms of health and disease. For instance, must one live full-time in a high amenity area, or will periodic visits confer some of the benefits? Second, they may inform personal decisions about where and how to live. Third, employers seeking to recruit and support a healthy workforce may consider these factors in choosing where to site facilities. Finally, these analyses may influence public debate about where to encourage residential development, how to allocate public lands, how to conserve natural ecosystem services, the routing of highways and ecological/biodiversity corridors, and other health and housing policy issues.

### Limitations

As with all non-randomized data, the possibility of confounding by unmeasured factors limits our ability to discern causality. In addition, these data cannot eliminate selection bias in which thinner, healthier people preferentially migrate to areas with greater natural amenities, perhaps to take advantage of outdoor recreation. Likewise, heavier people with more health problems may migrate to areas with lower amenities for medical care. However, the negative association of natural amenities with obesity persists while controlling for recreational facilities [9]. Sampling bias is a possibility, although the broad use of driver’s licenses by adults, plus the inclusion of non-driver identity cards, suggests a very generalizable sample of adults in the seven states in the DMV data.

Although heights and weights were measured in the VDIS data, they depend on self-report in the other data sets. It is highly likely that these data underestimate the prevalence of obesity as there is a systematic tendency for people to underestimate their own weight and overestimate their own height [17-18]. However, there is little reason to believe that this error is associated with natural amenities, meaning that it is unlikely to be influencing estimates of the association between amenities and obesity.

The ERS Natural Amenities Scale was developed primarily to study rural migration patterns and economic development and is well-suited to that purpose [7]. However, it includes only a subset of the natural environment factors that might influence obesity and includes no characterization of the built environment. Nonetheless, it is a robust predictor of obesity in this and other analyses, indicating that climate and topography are important correlates of obesity.

This analysis is limited to adults. Although much of the American landscape is represented, the data are not uniformly distributed across the country in all three data sets. Because the Natural Amenity Scale is not available for Hawaii and Alaska, they were not included in any of the analyses. Generalizability to children or other regions of the world is very uncertain.

The county-level covariates, including the Natural Amenities Scale, were collected in the mid-2000s, but the individual data were collected over a greater period of time. Natural amenities generally change very little over time, but changes in social and economic characteristics do, possibly adding error. However, we saw no major changes in the estimates of the relationship of natural amenities to obesity in the sub-analyses by decade.

Obesity as a complex phenomenon with contributions from diet, activity, genetics, and a multitude of other factors. Rather than provide a comprehensive analysis of all the causes of obesity, the logistic models were designed to examine the effects of potential confounders on the relationship between the ERS Natural Amenities Scale and obesity. The strength of association of the potential confounders (age, sex, census characteristics, etc.) to obesity was not of primary interest. Likewise, because they include only a few of the potential causative factors, the logistic regression models account for only a small proportion of the variance in subject-to-subject obesity, as reflected by low values for R^2^. 

## Conclusions

This is the largest analysis to date of the relationship of natural environmental factors to obesity. The very large and generalizable sample and the robustness of the main effects across various subgroups support the conclusion that residing in areas with access to natural amenities, such as open water and a variety of topographic features as well as cool, dry summers and warm, sunny winters, is associated with lower rates of obesity.
